# Supplementary material for: Lift&Add—rapid and robust addition of new species to alignments of conserved non-coding sequences
Source: Bioinformatics. 2026 May 27;42(6):btag315. doi: 10.1093/bioinformatics/btag315 (PMC13224966; doi:10.1093/bioinformatics/btag315)
Supplement: btag315_Supplementary_Data [file btag315_supplementary_data.zip › SupplementaryFigure_ShuklaGallegoRomero.pdf]

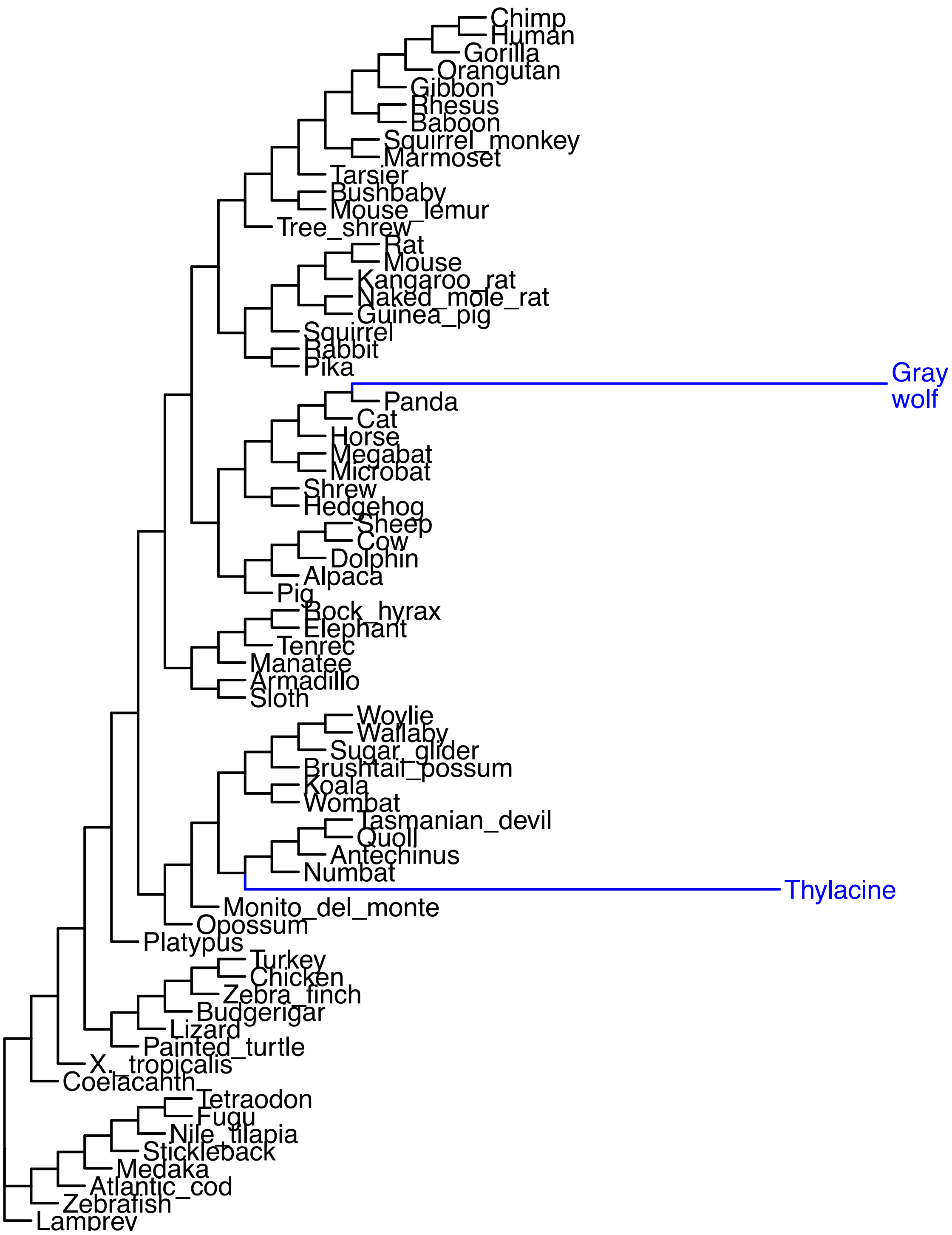

Supplementary Figure 1. Binary trait tree. Used as input in the RERConverge trait association analysis. In this tree, the foreground convergent lineages have branch lengths of 1 while the background lineages have branch lengths of 0.

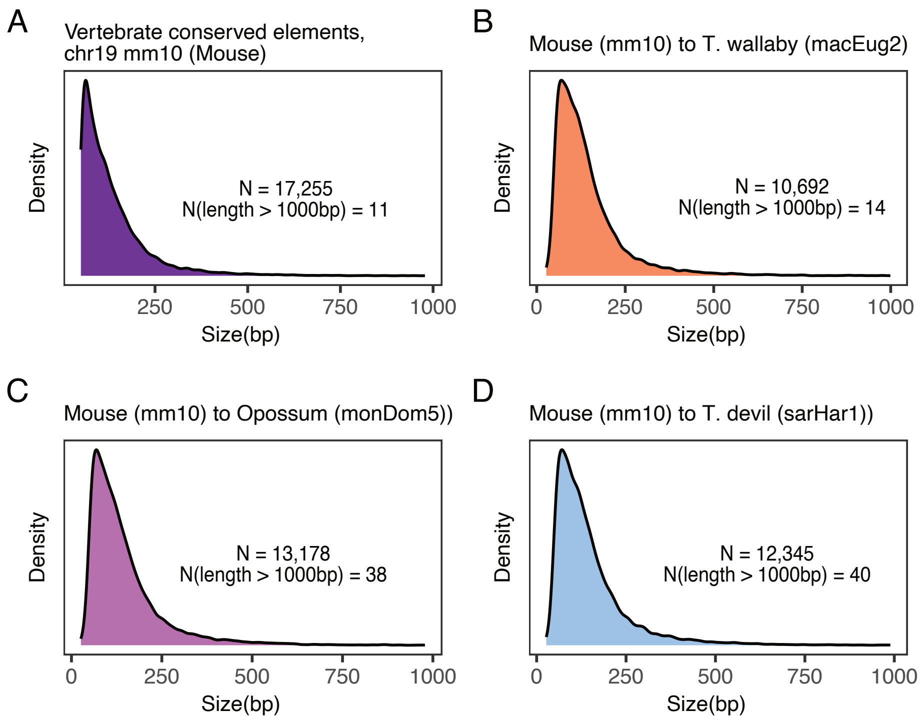

Supplementary Figure 2. Size (bp) of vertebrate conserved elements A. as estimated by phastCons from the 60-way whole-genome alignment, with output element coordinates corresponding to the reference mouse (mm10) genome. Size of vertebrate conserved elements, after liftOver from mouse to the **B.** tammar wallaby (macEug2) genome, **C.** gray short-tailed opossum (monDom5) genome and **D.** Tasmanian devil (sarHar1) genome.

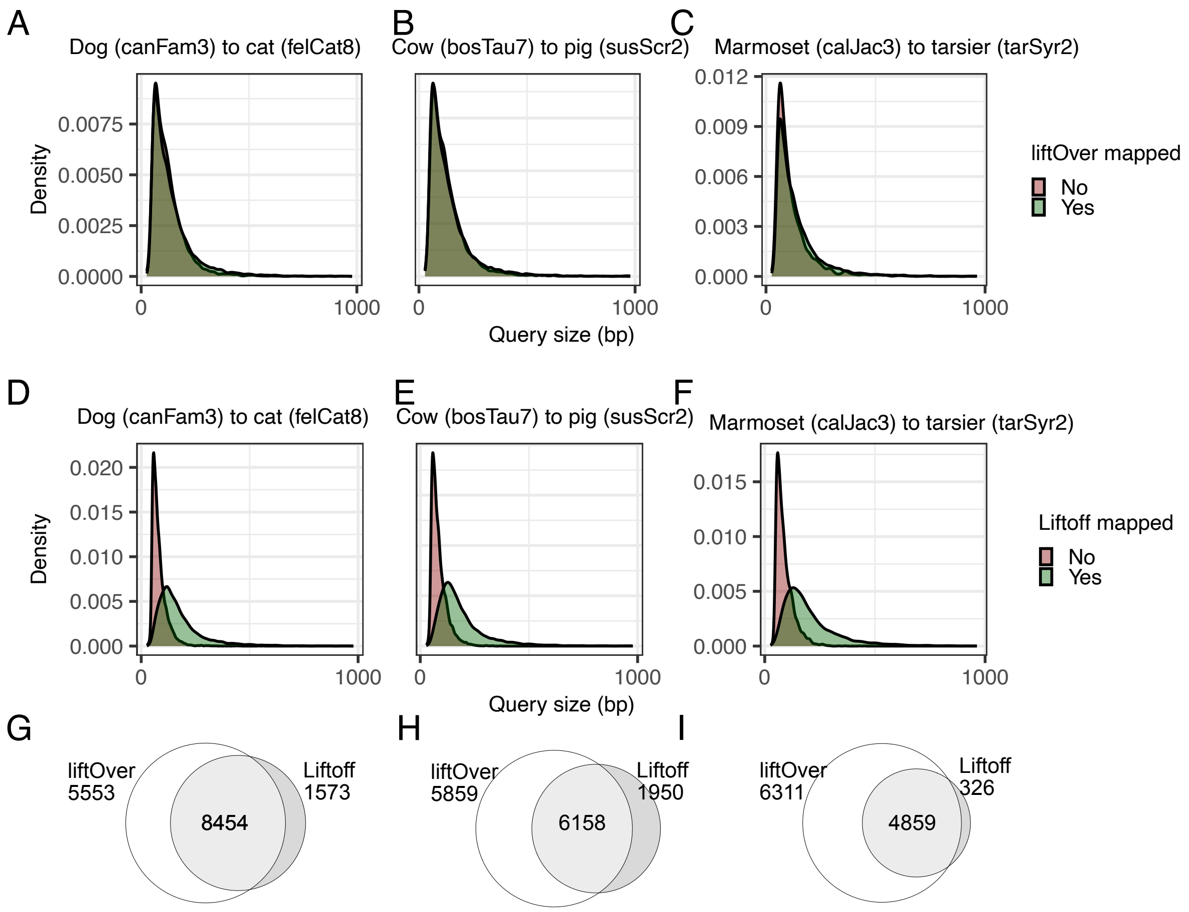

Supplementary Figure 3. Comparison of the size distribution and number of elements that were mapped with liftOver and Liftoff from **A, D, G** the dog (canFam3) to the domestic cat (felCat8) genome, **B, E, H** the cow (bosTau7) to the pig (susScr2) genome, and **C, F, I** the marmoset (calJac3) to the tarsier (tarSyr2) genomes. For size comparisons, elements > 1000 bp in size have been excluded.

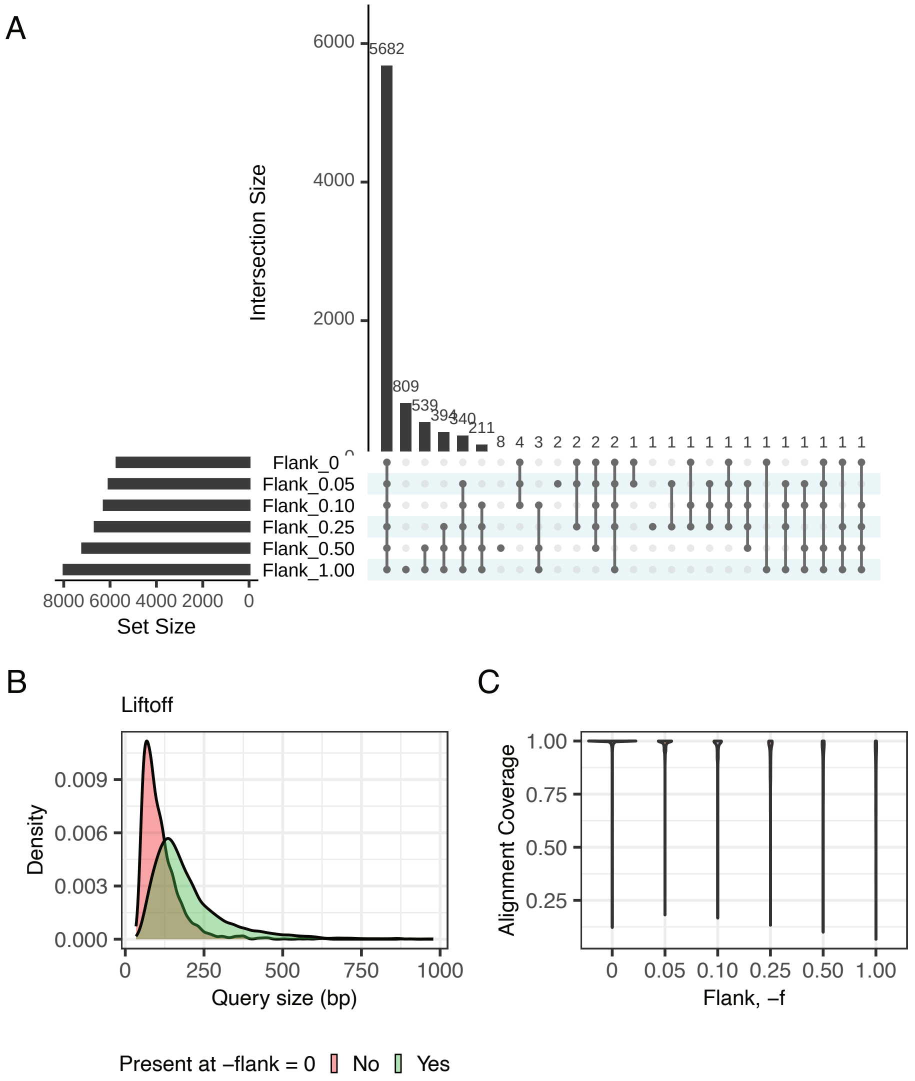

Supplementary Figure 4. **A.** Intersection of Liftoff output at different thresholds of `-flank` (0, 0.05, 0.10, 0.25, 0.50 and 1). **B.** Distribution of sizes for opossum query elements that are mapped to devil at `-flank` = 0 or `-flank` > 0. **C.** Distribution of alignment coverage for Liftoff output elements at different threshold of `-flank`.

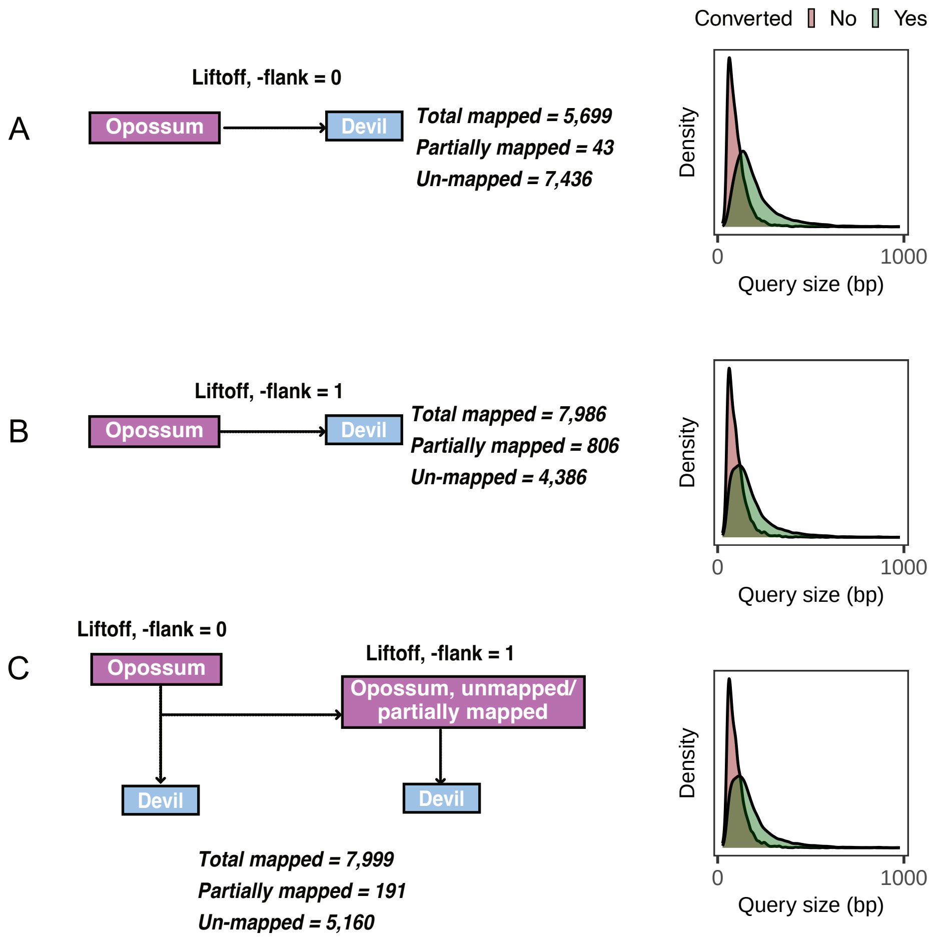

Supplementary Figure 5. Two-rounds of Liftoff to increase recovery and reduce partial mapping Summary of Liftoff output with **A.** No flanking genomic sequence added (`-flank` = 0), **B.** Maximum amount of flanking genomic sequence added (`-flank` = 1) and **C.** `-flank` = 0, followed by a second Liftoff for unmapped elements with `-flank` = 1.

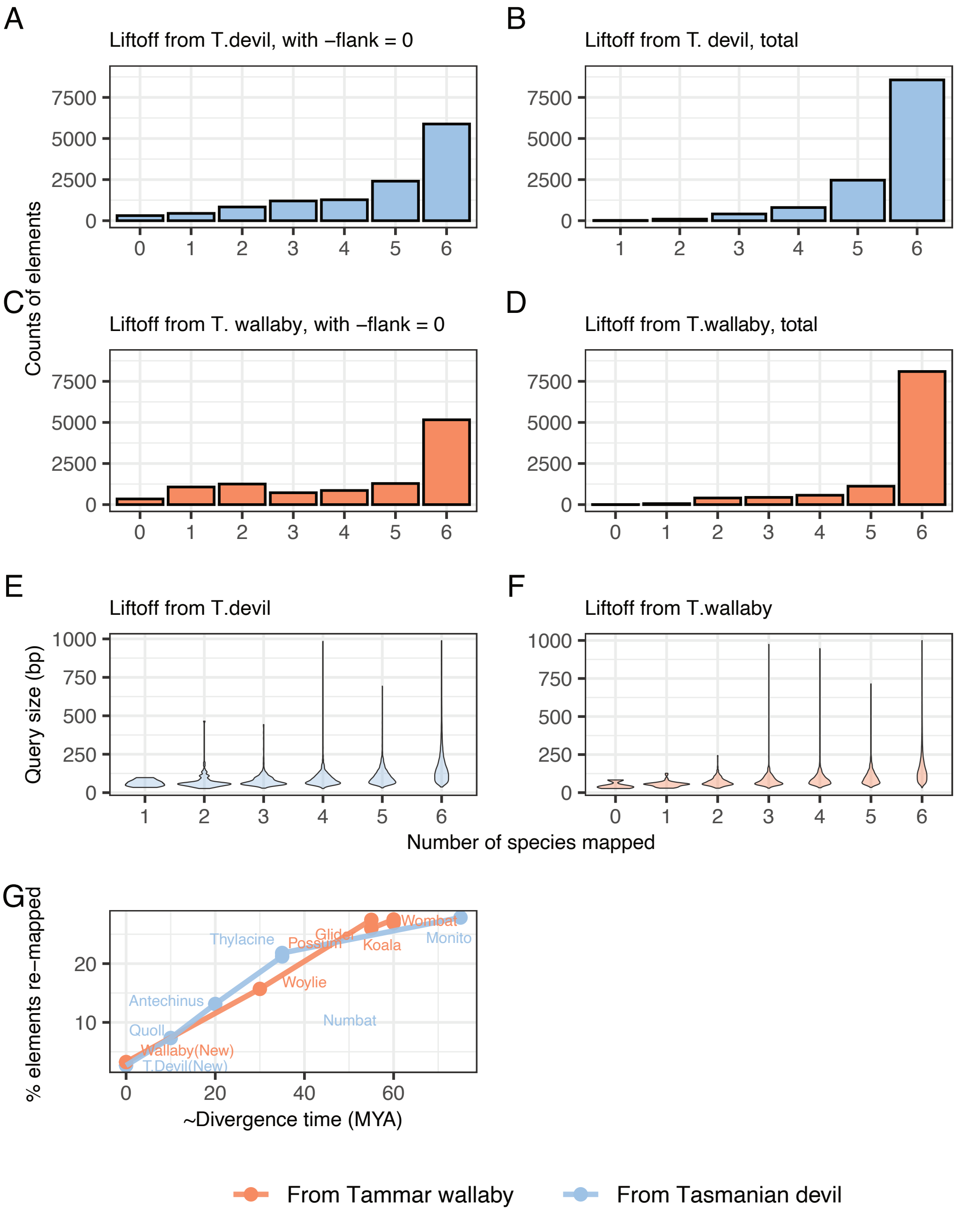

Supplementary Figure 6. Mapping elements with Liftoff from the Tasmanian devil and tammar wallaby (macEug2) to target marsupial genomes. A and C. Counts of query elements that were mapped to 0 or more species with the initial Liftoff using `-flank = 0`. **B and D.** Counts of query element that were mapped to 0 or more species, with both rounds of Liftoff (`-flank = 0`, followed by `-flank = 1` for unmapped elements) . **E and F.** Size distribution for query elements that mapped to 0 or more species after both rounds of Liftoff. **G.** Correlation between estimated divergence between query and target and the percentage of elements that mapped only with the addition of flanking sequence (`-flank` =1).

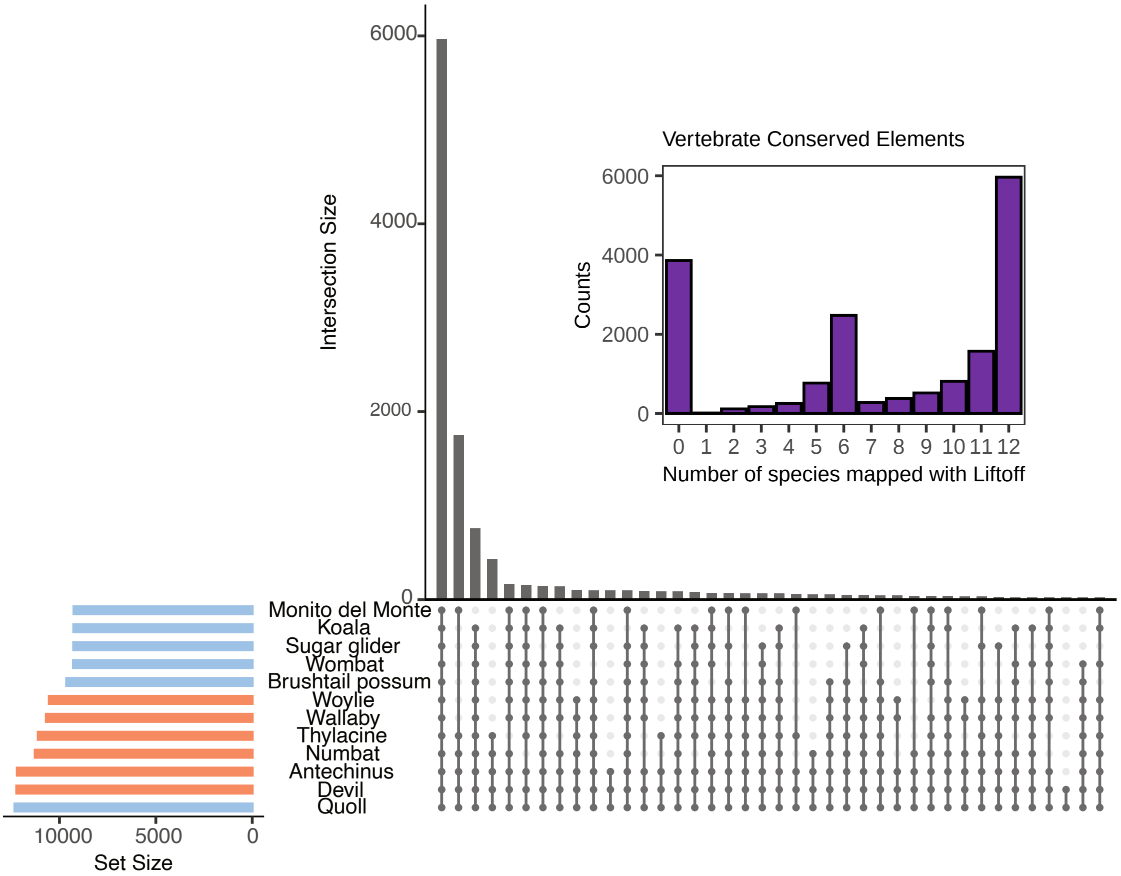

Supplementary Figure 7. Intersection of Liftoff output across the 12 marsupial target genomes. The histogram displays the number of the original 17,255 vertebrate conserved elements that were mapped to 0 or more species. The Dasyuromorphia taxa (and the monito del monte) are in blue and the Diprotodontia taxa are in orange.

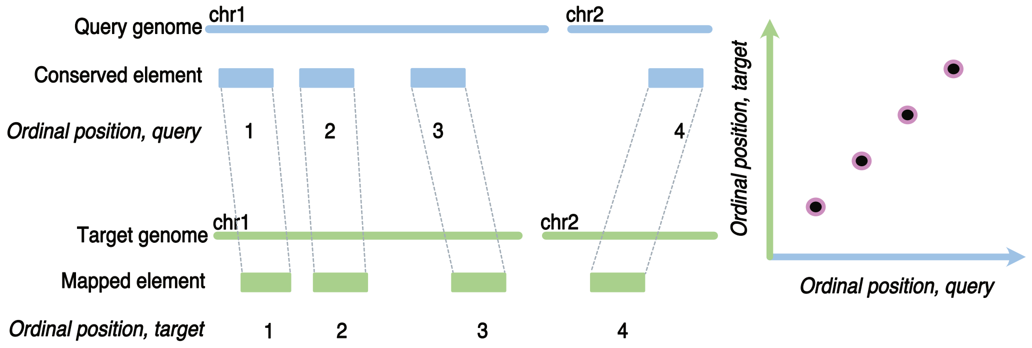

Supplementary Figure 8. Comparing co-linearity. A visual summary of our approach comparing co-linearity of conserved elements between the query and target genomes. Conserved elements were ordered by their position in the query genome, and once mapped, they were ordered by their position in the target genome. The correlation between these ordinal positions was then assessed.

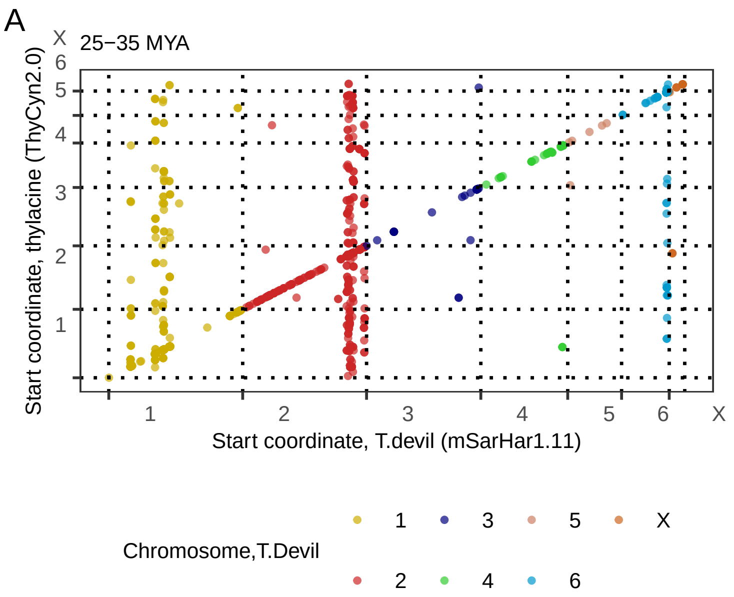

Supplementary Figure 9. Correlation in genomic coordinates of mapped elements between the Tasmanian devil (mSarHar1.11) and Thylacine (ThyCyn2.0), highlighting regions at which conserved elements are displaying variable mapping between these genomes.

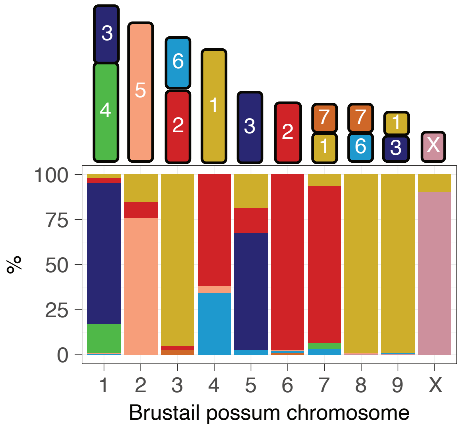

Supplementary Figure 10. Relationship between the tammar wallaby and brushtail possum karyotypes, as determined by [48], and proportion of test elements mapping to each brushtail possum chromosome, coloured by their location in the tammar wallaby genome.

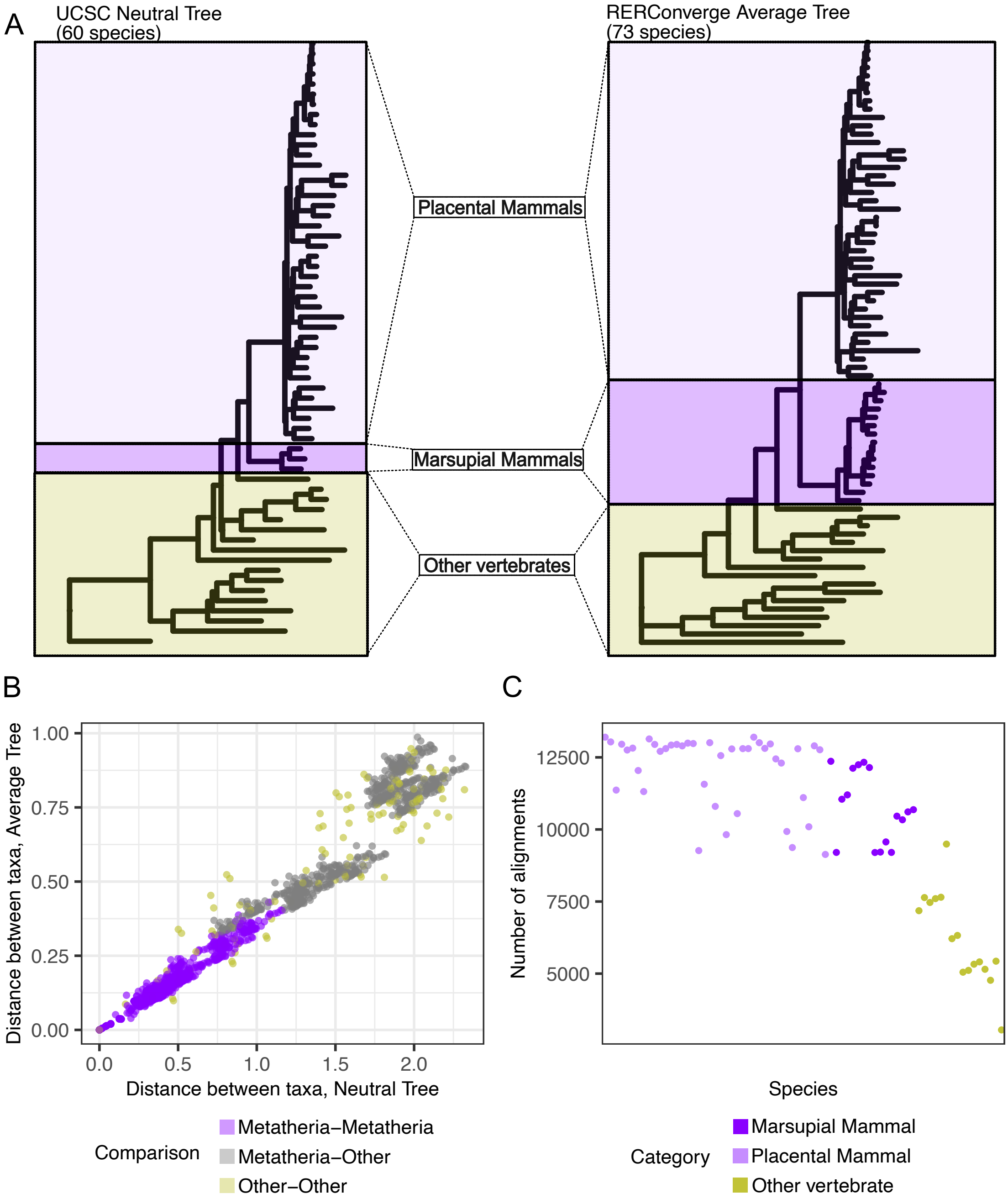

Supplementary Figure 11. Average tree, RERConverge. A. Neutral model of evolution for the 60 species WGA (left) and consensus tree estimated from alignments of 13,220 vertebrate conserved elements by RERConverge (right). **B.** Distances between terminal nodes in the neutral tree versus those in the average tree. **C.** Number of alignments (out of 13,220) in which each species is present.

Supplementary Tables

Supplementary Table 1: List of query genomes for Liftoff.

Supplementary Table 2: liftOver and Liftoff of vertebrate conserved elements from the gray short-tailed opossum genome (monDom5) to the Tasmanian devil genome (sarHar1).

Supplementary Table 3: Liftoff output at different threshold of `-flank`.

Supplementary Table 4: List of target marsupial assemblies.

Supplementary Table 5: Mapping results with Lift&Add for other pairs of mammalian (non-marsupial) query and target genomes.

Supplementary Table 6: Number of test elements from Wallaby chromosomes that mapped to each Brushtail possum chromosome with Liftoff.

Supplementary Table 7: Pairwise t-tests' (with a Benjamini-Hochberg correction) comparing alignment distances to the Tasmanian devil (sarHar1).

Supplementary Table 8: Pairwise t-tests' (with a Benjamini-Hochberg correction) comparing alignment distances to the tammar wallaby (macEug2).
